# Associations between the quality of life in sarcopenia measured with the SarQoL® and nutritional status

**DOI:** 10.1186/s12955-020-01619-2

**Published:** 2021-01-22

**Authors:** Yongtaek Kim, Ki Soo Park, Jun Il Yoo

**Affiliations:** 1 Public Health Medical Service Office, Chungnam National University Hospital, Daejeon, Republic of Korea; 2 Department of Preventive Medicine, Institute of Health Sciences, College of Medicine, Gyeongsang National University School of Medicine, Jinju, Republic of Korea; 3 Center for Farmer’s Safety and Health, Gyeongsang National University Hospital, Jinju, Republic of Korea; 4 Department of Orthopedic Surgery, Gyeongsang National University Hospital, 90 Chilamdong, Jinju, Republic of Korea

**Keywords:** Quality of life, Sarcopenia, Nutritional status, SarQoL

## Abstract

**Background:**

The purpose of this study was to evaluate the relationship between nutritional status and health-related quality of life after adjusting for essential factors of muscle mass, calf circumference, grip strength, and the timed up and go (TUG) test for diagnosis of sarcopenia.

**Methods:**

The subjects of this study were those who visited a health care center or a senior welfare center among the aged 65 years or older living in a community in two counties (Jinju, Sacheon), and the survey was conducted from April to August 2019. Among them, those with cardiovascular disease, cognitive disorder, or malignancy were excluded. To determine the nutritional status of the elderly subjects, a questionnaire-based screening tool called DETERMINE was used. Developed as a health-related quality of life tool for sarcopenia, the Sarcopenia-specific Quality of Life (SarQoL) questionnaire was used. For screening of sarcopenia, a rapid questionnaire based on self-reported information about falls, mobility, and strength known as the SARC-F questionnaire was used. Assessment of sarcopenia included skeletal muscle mass, calf circumference, grip strength, and the TUG test.

**Results:**

A total of 324 elderly people living in rural villages who were able to move to senior and welfare centers was surveyed. As a result of evaluating the association between SarQoL and nutritional risk in elderly subjects, the association was statistically significant in the moderate-risk group (B = − 5.542, *p* = 0.001) and in the high-risk group (B = − 8.136, *p* < 0.001) in comparison to the low-risk group. Significant correlations were found in all seven domains of SarQoL, except the fear domain.

**Conclusions:**

This study confirms an association between quality of life dimensions surveyed by the SarQoL questionnaire and nutritional status in elderly subjects. Therefore, appropriate interventions are needed following brief evaluation of sarcopenia and nutritional deficiency among elderly people in communities.

## Background

Recently, sarcopenia in older people has become an important health issue. Skeletal muscle weakness due to sarcopenia has been reported to increase mortality [1] and reduce quality of life [2–4]. For this reason, in 2016, the World Health Organization (WHO) officially identified sarcopenia as a disease of the elderly [5, 6]. In addition, sarcopenia has been classified as an important intervention target for elderly populations among medical staff and national health policies.

The important thing in old age life is not about the length of the remaining life, but about the quality of life. The quality of life of the elderly is the ability to achieve a satisfying, meaningful and satisfying life [7]. Sarcopenia in the elderly is associated with some adverse clinical outcomes such as physical impairment, limitation of mobility, decreased quality of life, increased risk of falls, hospitalization and mortality [8].

Older age may be the most important among numerous reported risk factors, but household status, lifestyle, physical inactivity, poor nutritional and dental status, and diseases (osteoporosis, metabolic diseases, etc.) were also independently associated with sarcopenia [9]. In particular, the likelihood of developing sarcopenia is significantly correlated with the number of cardiometabolic risk factors, notably diabetes, hypertension, and dyslipidemia. The pathogenesis of sarcopenia may involve satellite cell senescence, loss of motor neurons, less active neuromuscular junctions, hormonal status, proinflammatory cytokines, decreased mitochondrial function, abnormal myokine production, and weight loss accompanying decreased appetite [9].

To date, most studies have evaluated improvement of nutritional disorders evaluated by clinical outcomes (muscle mass, muscle strength, physical function, and blood tests) following interventions such as improved nutrition and exercise. Few studies have evaluated HRQOL. Moreover, the recently developed SarQoL questionnaire has been mostly utilized for reliability and validity verification. In other words, there are not a lot of studies that investigated the relationship between risk factors or components of sarcopenia (muscular strength, muscle mass, physical function, and other aspects) and HRQOL [10].

Therefore, the purpose of this study was to evaluate the relationship between nutritional status and HRQOL after adjusting for essential factors of muscle mass, calf circumference, grip strength, and the timed up and go (TUG) test for diagnosis of sarcopenia.

## Methods

### Study population

The subjects of this study were those who visited a health care center or a senior welfare center among the aged 65 years or older living in a community in two counties (Jinju, Sacheon), and the survey was conducted from April to August 2019. Among them, those with cardiovascular disease, cognitive disorder, or malignancy were excluded.

All surveys were conducted on a one-on-one basis after trained researchers interviewed subjects, explained the content of the survey, and obtained written informed consent from the subjects. In addition, this study was approved by the Institutional Review Board of Gyeongsang National University (Approval Number: GIRB-A19-0031).

### Materials

Demographic characteristics for investigation were sex, age, living alone, educational level, smoking, and patient health questionnaire-2 (PHQ-2) survey for depressive symptoms that were thought to be associated with sarcopenia and nutritional status. After the survey, the education level was categorized into primary school level and more than only primary school level. In addition, those with a score of 3 or more on the PHQ-2 were considered to likely have depressive symptoms [11].

### Assessment of nutritional status

To determine the nutritional status of elderly participants, a questionnaire screening tool called DETERMINE was used [12]. This auto-administered questionnaire consists of 10 questions, with the name, DETERMINE, referring to an alphabetic collection of the first letters (in English) of each item assessed. Thus, questionnaire items consist of Disease, Eating poorly, Tooth loss, Mouth pain, Economic hardship, Reduced social contact, Multiple medicines, Involuntary weight loss or gain, Need for assistance in self-care, and Elderly [people] over 80 years old.

Weights are assigned according to importance. Nutritional risk scores were divided into 0–21 points, with 0–2 points categorizing the low-risk group, 3–5 points for the moderate-risk group, and 6 points for the high-risk group.

### SarQoL questionnaire

Specialized questionnaire tools have been developed and used to assess health-related quality of life (QOL) in older people as their muscle strength deteriorates [2]. The SarQoL questionnaire consists of seven domains and assesses quality of life in physical, mental, and social areas in the context of sarcopenia [2, 3]. This questionnaire is used to assess quality of life following intervention for sarcopenia, as well as health-related QOL in living with the condition.

Developed as a health-related quality of life tool for sarcopenia, the SarQoL questionnaire consists of 55 items and 22 questions. The questionnaire calculates a total 7 domain scores and a total score, each between 0 and 100 points.

Domain score are 0–100, items are (mostly) likert scales. The domains (enumerated 1–7) and items are as follows: (1) physical and mental health, assessed with eight items, such as loss of arm strength or feelings of being frail, (2) locomotion, assessed with nine items, such as limitations in walking time and difficulty in walking on uneven ground, (3) body composition, assessed with three items, such as loss of muscle mass, (4) functionality, assessed with 14 items, such as balance problems and frequency of falls, (5) activities of daily living, assessed with 15 items, such as difficulty during light physical effort, fatigue during light physical effort, and use of public transportation, (6) leisure activities, assessed with two items, such as changes in leisure activities, and (7) feelings of fear, assessed with four items, such as fear of getting hurt and fear of falling [3, 13].

### Sarcopenia variables

The SARC-F questionnaire was developed for screening sarcopenia in the elderly. Question items consist of five categories of strength, assistance required for walking, rising from a chair, climbing stairs, and frequency of falls. Sarcopenia is suspected in patients receiving 4 points or more.

Calf circumference qualified participants for the sarcopenia (robust vs low) risk group with a cut-off point less than 34 cm for men and less than 33 cm for women [9].

The skeletal muscle index (SMI) was calculated using the following bioelectrical impedance analysis (BIA) regression equation (measured by InBody 720, Biospace Co., Ltd., Seoul, Korea): skeletal muscle (kg) = [0.401 × (height^2^/resistance) + (3.825 × sex) − (0.071 × age) + 5.102]. Low muscle mass was classified as a height-adjusted SMI value < 5.7 kg/m^2^ for women and < 7.0 kg/m^2^ for men [9].

Grip strength was measured using a smedley dynamometer (TK 5001 Grip-A, Takei, Tokyo, Japan), and dominant hand grip strength was classified as “robust” if the value was over 28 kg for men and over 18 kg for women [9].

To measure physical performance using the timed up and go (TUG) test, each individual was asked to stand from a sitting position, walk 3 m, and then return to a sitting position on the same chair, during which a trained researcher measured the time (in seconds) required to perform this task. Times were divided into robust and low level based on a baseline of 10 s [14].

### Statistical analysis

The results were expressed as the median (percentile, 25 and 75) for those that did not followed a normal distribution. The normality of the variables was checked by a Kolmogorov–Smirnov test. Frequencies (N, %) were described for qualitative variables.

Logistic regression analysis was performed to determine the association between nutritional status and SARC-F. Multiple regression analysis was performed to determine the association between SarQoL and nutritional intake, sarcopenia variables. Nutritional status was included in the analysis as a dummy variable based on a baseline of good. Two statistical analyses were performed after adjusting for demographic characteristics (sex, age, living alone, educational level, smoking) and symptoms of depression that were thought to be associated with sarcopenia and nutritional status. Statistical program SPSS 25.0 was used, and a *p* value less than 0.05 was considered statistically significant.

## Results

### General characteristics of participants

Study participants comprised 324 individuals living in six rural villages, and research was conducted at senior and welfare centers. All participants aged 65 years who lived in villages and were able to move to senior and welfare centers were surveyed. Of a population of 351 elderly people, 341 responded that they did not suffer from cognitive impairment, cancer, stroke, or myocardial infarction. A total of 324 participants was finally included in the investigation (341 healthy respondents, excepting 17, who declined to consent to participation).

The median (percentile 25 and 75) age of study subjects was 72.0 (68.3, 78.0) years, with women comprising 283 (87.3%) of total subjects. Of the elderly subject sample, 47.5% lived alone and 31.2% had depression. Skeletal muscle mass, calf circumference, grip strength, and TUG decreased in 11.1%, 43.5%, 21.9%, and 30.9% of all subjects, respectively. In terms of nutritional assessment, the low-risk group comprised 42.6%, the moderate-risk group comprised 29.3%, and the high-risk group comprised 28.1% of the sample. The overall (median (percentile of 25, 75)) of SarQoL was 70.2 (56.0, 81.8), among the sub-domains, physical and mental health had the lowest quality of life scores (62.2 (51.4, 76.6)), and fears were the highest (87.5 (75.0, 100.0)) (Table 1).

**Table 1:** General characteristics of participants

Variables		N (%)
Sex	Male	41 (12.7)
	Female	283 (87.3)
Age	Median (25%, 75%)	72.0 (68.3, 78.0)
Living alone	No	170 (52.5)
	Yes	154 (47.5)
Education level	≤ 6 years	196 (60.5)
	≥ 7 years	128 (39.5)
Smoking	No	314 (96.9)
	Yes	10 (3.1)
Depression	No	223 (68.8)
	Yes	101 (31.2)
SARC_F	< Probable not sarcopenia	256 (79.0)
	≥ Probable sarcopenia	68 (21.0)
SMI	Robust	288 (88.9)
	Low	36 (11.1)
Calf circumference	Robust	183 (56.5)
	Low	141 (43.5)
Grip strength	Robust	253 (78.1)
	Low	71 (21.9)
TUG	Robust	224 (69.1)
	Low	100 (30.9)
Determine (range: 0–21)	0–2	138 (42.6)
	3–5	95 (29.3)
	≥ 6	91 (28.1)
SalQoL		
Physical and mental health	Median (25%, 75%)	62.2 (51.4, 76.6)
Locomotion	Median (25%, 75%)	66.7 (50.0, 91.7)
Body composition	Median (25%, 75%)	62.5 (50.0, 79.2)
Functionality	Median (25%, 75%)	73.2 (57.7, 86.5)
Activities of daily living	Median (25%, 75%)	70.0 (55.4, 85.0)
Leisure activities	Median (25%, 75%)	66.5 (33.3, 66.5)
Fears	Median (25%, 75%)	87.5 (75.0, 100.0)
Overall	Median (25%, 75%)	70.2 (56.0 81.8)
Total		324 (100.0)

TUG, timed up and go; SMI, skeletal muscle index; DETERMINE: Disease, Eating poorly, Tooth loss, Mouth pain, Economic hardship, Reduced social contact, Multiple medicines, Involuntary weight loss or gain, Need for assistance in self-care, and Elderly; SarQoL, Sarcopenia-specific Quality of Life

### Association between SARC-F and nutritional status

To evaluate the association between SARC-F and sarcopenia assessment, nutritional status, logistic regression analysis was performed. Among the assessment tools for evaluating sarcopenia, calf circumference (OR = 2.150, 95% CI = 1.057–4.376, *p* = 0.037) and TUG (OR = 3.414, 95% CI = 1.710–6.816, *p* = 0.001) showed statistically significant (Table 2). SARC-F and nutritional status (Determine ≥ 6 vs. < 3) were statistically associated (OR = 3.703, 95% CI = 1.580–8.682, *p* = 0.003).

**Table 2:** Association between SARC_F (suspected sarcopenia (≥ 4) versus not suspected sarcopenia (< 4)) and nutritional status

Dependent variable: SARC_F	OR	95% CI for OR		*P* value
		Lower	upper	
SMI (ref: robust)	1.218	0.470	3.156	0.685
Calf circumference (ref: robust)	2.150	1.057	4.376	0.035
Grip strength (ref: robust)	1.944	0.957	3.950	0.066
TUG (ref: robust)	3.414	1.710	6.816	0.001
Determine (score: 3–5)	1.798	0.768	4.206	0.176
Determine (score ≥ 6)	3.703	1.580	8.682	0.003

Adjusted for demographic characteristics (sex, age, living alone, education level, smoking) and depressive symptoms

OR, odds ratio; TUG, timed up and go; SMI, skeletal muscle index; Determine: Disease, Eating poorly, Tooth loss, Mouth pain, Economic hardship, Reduced social contact, Multiple medicines, Involuntary weight loss or gain, Need for assistance in self-care, and Elderly

### Relationship between SarQoL domain and nutritional status

Total score of SarQoL was significant in relationship to SARC-F (B = − 13.314, *p* < 0.001), grip strength (B = − 3.866, *p* = 0.034), and TUG (B = − 5.459, *p* = 0.001). The relationship between total score of SarQoL and nutritional status was significant in the moderate-risk group (B = − 5.542, *p* = 0.001) and in the high-risk group (B = − 8.136, *p* < 0.001) in comparison to the low-risk group (Table 3).

**Table 3:** The relationship between overall score of SarQoL and nutritional status

Dependent variable: overall score of SarQoL	B	SE	95% CI for B		*P* value
			Lower	Upper	
SARC_F (ref: < 4)	− 13.314	1.900	− 17.053	− 9.575	< .001
SMI (ref: robust)	.002	2.335	− 4.593	4.597	.999
Calf circumference (ref: robust)	.411	1.532	− 2.603	3.425	.789
Grip strength (ref: robust)	− 3.866	1.820	− 7.448	− .285	.034
TUG (ref: robust)	− 5.459	1.682	− 8.768	− 2.150	.001
Determine (score: 3–5)	− 5.542	1.666	− 8.821	− 2.264	.001
Determine (score: ≥ 6)	− 8.136	1.882	− 11.839	− 4.433	< .001

Adjusted for demographic characteristics (sex, age, living alone, education level, smoking) and depressive symptoms

OR, odds ratio; TUG, timed up and go; SMI, skeletal muscle index; Determine, Disease, Eating poorly, Tooth loss, Mouth pain, Economic hardship, Reduced social contact, Multiple medicines, Involuntary weight loss or gain, Need for assistance in self-care, and Elderly; SarQoL, Sarcopenia-specific Quality of Life

Relationships between physical and mental health and nutritional status were statistically significant in the moderate-risk group (B = − 4.353, *p* = 0.033) and in the high-risk group (B = − 7.013, *p* = 0.002) in comparison to the low-risk group. Trends were shown in locomotion (moderate-risk group (B = − 6.127, *p* = 0.026) and high-risk group (B = − 14.801, *p* < 0.001)), body composition (moderate-risk group (B = − 4.924, *p* = 0.019) and high-risk group (B = − 5.670, *p* = 0.017)), functionality (moderate-risk group (B = − 5.542, *p* = 0.012) and high-risk group (B = − 7.480, *p* = 0.003)), activities of daily living (moderate-risk group (B = − 5.857, *p* = 0.002) and high-risk group (B = − 5.287, *p* = 0.014)), and leisure activities (moderate-risk group (B = − 8.662, *p* = 0.004) and high-risk group (B = − 14.943, *p* < 0.001)). In particular, leisure activities were only significantly related to nutritional status. However, SARC-F was significantly related to physical and mental health (B = − 11.741, *p* < 0.001), locomotion (B = − 16.935, *p* < 0.001), body composition (B = − 9.124, *p* < 0.001), functionality (B = − 13.897, *p* < 0.001), and activities of daily living (B = − 14.336, *p* < 0.001). Findings from TUG tests were significantly related to locomotion (B = − 8.010, *p* = 0.001), functionality (B = − 6.869, *p* = 0.002), and activities of daily living (B = − 7.210, *p* < 0.001). In addition, grip strength was significantly related to activities of daily living (B = − 5.103, *p* = 0.014) and feelings of fear (B = − 7.243, *p* = 0.001). However, there were no SarQoL domains related to limb muscle mass or calf circumference.

## Discussion

As a result of evaluating the relationships between SarQoL domains and nutritional risk questionnaire findings in elderly subjects, significant correlations were found in seven domains of SarQoL, with the exception of the fear domain. The domain of leisure activity is not shown to be related to the variables assessing sarcopenia, but only to nutritional risk assessment. Overall SarQoL score was associated with SARC-F, TUG, nutritional risk.

Malnutrition is documented in up to 50% of older adults, but rates of prevalence vary considerably based on demographics studied, health environment, and the method used to measure it [15, 16]. A recent systematic review and meta-analysis of studies using the Mini Nutritional Assessment shows that a high proportion of older adults are at risk of malnutrition, with estimates ranging from 27% (community/outpatient settings) to 50% (all other healthcare settings) [15]. In the present study, the risk of malnutrition in participants was 29.3% (moderate) and 28.1% (severe). Therefore, it is estimated that more than 50% of rural-dwelling elderly in a community setting are malnourished. In particular, older people in East Asia, including Korea, eat rice and vegetables as dietary staples, which can contribute to malnutrition and protein deficiency [17, 18]. Consequently, these malnourished subjects are at increased risk of sarcopenia due to lack of protein intake.

In addition, the depression used as an adjusted variable was also significantly associated with SarQoL (data not shown). The results are the same as the study [19] that showed more depression when the muscle mass or strength were insufficient. Brain atrophy that may develop and become depressed when sarcopenia progresses [20], and chronic inflammation and nutritional deficiencies that cause sarcopenia are the causes of depression in the elderly [21]. Therefore, in the future, a longitudinal study is needed to determine whether or not depression occurs due to the presence or absence of sarcopenia.

The SARC-F, a rapid questionnaire based on self-reported information about falls, mobility, and strength, has been developed for early detection of sarcopenia [9, 22, 23]. Previous studies have shown similar results of association of malnutrition and sarcopenia [21, 24, 25]. In the lastest version of the diagnostic criteria for sarcopenia [9, 22], the SARC-F questionnaire first recommended finding at-risk groups for sarcopenia, followed by assessment of muscle strength and muscle mass and, finally, assessment of physical function to classify severity. Even after applying these guidelines, studies show that people suspected of malnutrition in communities are at increased risk of sarcopenia. In other words, proper interventions for malnourished elderly in communities can reduce sarcopenia. Although assessed by simple questions comprising tools such as DERTERMINE and SARC-F, most SarQoL domains quickly reveal poor sarcopenia-related quality of life, with the exception of fear items in the SarQoL. Such assessment also helps to identify a group of early interventions for sarcopenia. Leisure activities (e.g., dining out, gardening, furniture making, fishing, elderly-specific activities, and walking) have only been shown to be significantly associated with DETERMINE. To enjoy these specified leisure activities in rural areas of Korea, the economic status of elderly subjects must be good, and these people will have good nutritional status.

In particular, questionnaires such as DERTERMINE have been specially developed to identify nutritional deficiency in the elderly, and health-related quality of life is not expected to be good for people with moderate or severe risk of nutritional deficiency. For these populations, interventions must first be made, and nutritional interventions are required together with exercise.

## Limitations and further objectives

There are several limitations in this study. First, as a result of the cross-sectional study design, a causal relationship between nutritional status and SarQoL cannot be explained. In addition, the study subjects do not represent elderly people in the general population because only elderly people in rural areas were targeted. Second, further studies on factors affecting nutritional risks in rural-dwelling elderly people are needed, and intervention studies based on these results may be conducted. Third, laboratory tests were not analyzed to assess the current nutritional status.

## Conclusions

This study confirms an association between quality of life dimensions surveyed by the SarQoL questionnaire and nutritional status in elderly subjects. Therefore, appropriate interventions are needed following brief evaluation of sarcopenia and nutritional deficiency among elderly people in communities.

## Data Availability

Not applicable

## Abbreviations

HRQOL: Health-related quality of life
TUG: Timed up and go
SMI: Skeletal muscle index
DETERMINE: Disease, Eating poorly, Tooth loss, Mouth pain, Economic hardship, Reduced social contact, Multiple medicines, Involuntary weight loss or gain, Need for assistance in self-care, and Elderly
SarQoL: Sarcopenia-specific Quality of Life
WHO: World Health Organization
